# Clearing the Noise: Seasonal Dynamics of Endophytic Bacteria in *Fagus sylvatica* Leaves Revealed by Application of PNA Clamps

**DOI:** 10.1111/ppl.70897

**Published:** 2026-04-26

**Authors:** Irene Giubilei, Silvia Turco, Antonella Cardacino, Lovely Mahawar, Benedicte Riber Albrectsen, Angelo Mazzaglia

**Affiliations:** ^1^ Dipartimento di Scienze Agrarie e Forestali Università Degli Studi Della Tuscia Viterbo Italy; ^2^ Department of Plant Physiology, Umeå Plant Science Centre Umeå University Umeå Sweden

**Keywords:** endophytes, *Fagus sylvatica*, metabarcoding, microbiome, PNA clamps

## Abstract

The characterization of the seasonal dynamics of endophytic bacteria in beech leaves can be hindered by co‐amplification of chloroplast and mitochondrial plant DNA. This study applies established peptide nucleic acid (PNA) clamps to suppress host‐derived amplification while resolving bacterial succession across the vegetative season. Chloroplast‐ and mitochondrion‐specific PNAs inverted the proportion of host to bacterial reads, enabled the recovery of bacterial sequence variants, and increased alpha diversity accordingly. Beta‐diversity analyses showed that, once host contamination was removed, samples displayed a clear seasonal trajectory. Early‐season leaves contained high abundances of *Pseudomonas* together with taxa likely introduced through plant–insect–microbe interactions. As leaves matured, the microbiome shifted toward a more stable composition dominated by well‐established genera. The transition from early transient taxa to the later enrichment of phyllosphere‐adapted and nutrient‐cycling genera demonstrates that beech leaves host a temporally structured microbiome shaped by leaf development and seasonal environmental stress.

## 1 Introduction

The European beech ( *Fagus sylvatica* L.) is a key deciduous tree species widely distributed across Central and Western Europe, with a natural range stretching from Greece to Spain and from Sicily to Scandinavia (Durrant et al. 2016). Its ecological success is largely attributed to its capacity to thrive across a broad spectrum of soil types and altitudinal gradients (Liepiņš and Bleive 2025). However, in recent decades, beech forests have been increasingly affected by a range of biotic and abiotic stressors, including pathogenic microorganisms, prolonged drought, and rising temperatures, which are becoming more frequent and severe under ongoing climate change scenarios (Luchi et al. 2006).

At the same time, *F. sylvatica* hosts a diverse community of endophytic microorganisms, mainly fungi and bacteria, which colonize internal plant tissues and establish complex interactions with the host. The structure of these microbial communities is influenced by intrinsic factors such as host species and plant organ type, as well as by extrinsic variables, including climate and surrounding vegetation, all of which shape the diversity and dynamics of the endophytic microbiota (Siddique et al. 2021).

These endophytes are known to play crucial roles in forest ecosystems by supporting plant health, enhancing tolerance to environmental stress (Terhonen et al. 2023), promoting growth, and contributing to climate change resilience (Kivlin et al. 2013). Endophytic bacteria, in particular, have gained increasing attention for their potential applications in agriculture and forestry, especially as biocontrol agents and plant growth promoters (Ali et al. 2024; De Vries et al. 2020; Falade et al. 2021). Their beneficial effects are mediated through various mechanisms, including competition for space and nutrients, the production of lytic enzymes and secondary metabolites, and the activation of plant defense responses (Morales‐Cedeño et al. 2021; Oukala et al. 2021). Several bacterial genera, such as *Bacillus*, *Paenibacillus*, *Pseudomonas*, *Burkholderia*, *Enterobacter*, *Klebsiella*, and *Arthrobacter* are frequently reported as endophytes and have been studied for their roles in plant protection and development (Ahmad et al. 2022; Christakis et al. 2021; Christakis et al. 2021; Mowafy et al. 2021; Mutungi et al. 2022; Khan et al. 2020; Bolivar‐Anillo et al. 2021; Laforest‐Lapointe et al. 2017; Terhonen et al. 2019). In foliar tissues, however, the diversity and structure of these bacterial communities are not solely determined by their functional potential, but are strongly shaped by species‐specific leaf traits and local microclimatic conditions (Vacher et al. 2016). High‐throughput amplicon sequencing (HTS) currently represents the most widely used technique for analyzing microbial community composition in complex matrices such as plant tissues and soil (Viotti et al. 2024). However, a significant limitation of this approach when applied to plant‐associated microbiomes is the predominance of host‐derived DNA, especially chloroplast and mitochondrial sequences (Fitzpatrick et al. 2018; Lundberg et al. 2013; Dubois et al. 2025). Such overwhelming contamination can substantially reduce the resolution of microbial profiling and limit the detection of low‐abundance taxa (Giangacomo et al. 2021; Taerum et al. 2020). To address this issue, recent studies have employed peptide nucleic acid (PNA) clamps, synthetic oligonucleotides that bind selectively and stably to conserved regions of host organellar DNA, thereby blocking their amplification during PCR (Karkare and Bhatnagar 2006). By preventing the elongation of chloroplast and mitochondrial DNA, PNA clamps significantly reduce host read contamination and improve microbial sequence recovery, enhancing both the depth and accuracy of metabarcoding analyses (Lundberg et al. 2013). While universal PNAs can often be successfully applied across different host species when organellar sequences are conserved, as demonstrated in this study, specific design guidelines have been recently proposed for cases requiring host‐specific optimization (Dubois et al. 2025).

This approach has proven particularly valuable in plant microbiome studies, where standard primers often co‐amplify host DNA due to high sequence similarity with microbial targets (Giangacomo et al. 2021).

In this context, the present study aimed to investigate the composition and diversity of endophytic bacterial communities inhabiting the leaf tissues of *Fagus sylvatica* , harvested across seasons from one stand of trees. To achieve this, we employed 16S rRNA gene high‐throughput sequencing using the PNA clamps developed by Lundberg et al. (2013) to selectively target chloroplast and mitochondrial DNA. This strategy allowed us to limit host interference while preserving the ecological integrity of the microbial community. By enhancing taxonomic resolution, our findings offer new insights into the structure of beech‐associated endophytic communities and their evolution during the vegetative season, aiming to contribute to a broader understanding of their potential role in tree health and forest ecosystem resilience.

## 2 Materials and Methods

### 2.1 Sampling of Beech Leaves in Monte Raschio

Leaf sampling was conducted in 2024 in Monte Raschio (latitude 42°10′30″ N; longitude 12°09′16″ E), located in Oriolo Romano (Viterbo province, Central Italy), within the Bracciano–Martignano Natural Park. This area is part of the Special Area of Conservation (SAC) initiative “Faggete di Monte Raschio e Oriolo” (IT6010034). It reaches an elevation of ~500 m a.s.l., and includes a small, well‐preserved relic population of European beech ( *Fagus sylvatica* L.) considered ecologically distinctive. A total of five beech trees were selected for repeated leaf sampling at three time points during the growing season: TP1 (May 2024), TP2 (July 2024), and TP3 (October 2024), to characterize the temporal evolution of the bacterial endophytic community. At each time point, ~50 healthy‐looking leaves were collected per tree from five branches (10 leaves per branch). For each time point, 10 leaves of similar size and morphology were selected and pooled to generate one composite sample. Samples were immediately placed in sterile bags, kept on ice, and transported to the laboratory, where they were processed within 24 h.

### 2.2 DNA Extraction

Leaves were surface sterilised by sequential immersion in 70% ethanol (2 min), 1% sodium hypochlorite (3 min), and 70% ethanol (1 min), followed by three rinses with sterile distilled water. After air‐drying under sterile conditions, samples were kept at −80°C for 24 h, lyophilised for 48 h, and ground in liquid nitrogen.

Genomic DNA was extracted from 50 mg of powdered leaf material per sample using a modified CTAB protocol (Doyle 1991). DNA extraction was performed in two technical replicates per sample for each time point, resulting in a total of six extractions (*n* = 6). Briefly, each sample was mixed with 1 mL of extraction buffer [2% CTAB, 0.1 M Tris–HCl (pH 8.0), 20 mM EDTA, 1.4 M NaCl] in 2 mL microcentrifuge tubes and incubated at 65°C for 30 min. After a centrifugation step at 12,300*g* for 20 min, 1 mL of supernatant was transferred to a new sterile tube and mixed with 1 mL of chloroform/isoamyl alcohol (24:1) by gentle shaking. After centrifugation at 20,800*g* for 20 min, the aqueous phase was recovered, and DNA was precipitated with 0.6 volumes of cold isopropanol, incubating at −20°C for 1 h. The DNA pellet was collected by centrifugation (20,800*g* for 20 min), washed with 1 mL of 70% ethanol, air‐dried, and resuspended in 100 μL of nuclease‐free water. DNA yield was accurately determined using the Qubit dsDNA HS Assay Kit (Thermo Fisher Scientific), while DNA purity was assessed using an OPTIZEN NanoQ spectrophotometer (K LAB, Republic of Korea).

### 2.3 PNA Clamps to Block Host‐Plant Derived Amplification

To minimize host‐derived DNA amplification during bacterial 16S rRNA gene PCR, peptide nucleic acid oligomers (PNA clamps) targeting plant chloroplast and mitochondrial 16S rRNA gene regions were employed, as described by Lundberg et al. (2013). These PNA clamps selectively bind conserved plant‐derived sequences, thereby suppressing non‐bacterial amplification. The plastid‐specific PNA (pPNA: GGCTCAACCCTGGACAG) and the mitochondrial‐specific PNA (mPNA: GGCAAGTGTTCTTCGGA) were verified in silico against the *Fagus sylvatica* chloroplast and mitochondrial genomes (GenBank accession numbers OR936131.1 and MW771358.1; positions 88,000–91,250), showing 100% sequence identity with the respective target regions. PNA oligomers were synthesized by Eurogentec and stored at −20°C as 100 μM stock solutions and 10 μM working aliquots.

### 2.4 Mock Community to Assess PNA Effectiveness

As a mock community for comparison with the environmental samples, bacterial strains from the genera *Pseudomonas* (ID: UNITUS‐P28), *Erwinia* (ID: UNITUS‐E11), *Xanthomonas* (ID: UNITUS‐X89), and *Serratia* (ID: UNITUS‐S7) were obtained from the *in‐house* microbial collection of the Plant Pathology Laboratory at the University of Tuscia (Viterbo, Italy). Each isolate was cultured on yeast extract agar (YEA) plates at 28°C for 48 h. Genomic DNA was extracted from the resulting bacterial biomass using the NucleoSpin Plant II DNA Kit, following the manufacturer's instructions. DNA concentrations were measured with the Qubit dsDNA HS Assay Kit (Thermo Fisher Scientific), and defined amounts of DNA from each strain were pooled to obtain the final mock community: *Pseudomonas* (275 ng), *Erwinia* (100 ng), *Xanthomonas* (70 ng), and *Serratia* (15 ng).

### 2.5 16S rRNA Gene Amplification and Library Preparation

Bacterial community composition was assessed by targeting the V3–V4 region of the 16S rRNA gene, amplified from each DNA sample, including the mock community, using the primer pair 341F (5′‐TACGGGAGGCAGCAG‐3′) and 800R (5′‐CCAGGGTATCTAATCC‐3′) (Turner et al. 1999; Kisand et al. 2002). Both primers were synthesized with TruSeq Illumina adapters by Eurofins Genomics. PCR reactions were performed in a final volume of 25 μL containing 12.5 μL of 2× GoTaq G2 Master Mix (Promega), 1 μL of each primer (final concentration 0.4 μM), 1.25 μL of mitochondrial PNA (mPNA; final concentration 0.5 μM), 1.25 μL of plastid PNA (pPNA; final concentration 0.5 μM) as suggested by the PNA Bio technical guidelines (https://pnabio.com/), 2 μL of template DNA, and nuclease‐free water. Control reactions without PNA clamps (noPNA) were prepared under identical conditions, replacing PNAs with an equivalent volume of nuclease‐free water to maintain consistent reaction volumes and reagent concentrations. PCR amplifications were carried out using a GeneExplorer GE‐96G Thermal Cycler (Hangzhou Bioer Technology Co. Ltd) under the following cycling conditions: an initial denaturation at 95°C for 3 min, followed by 34 cycles of denaturation at 95°C for 30 s, PNA annealing at 78°C for 10 s, primer annealing at 55°C for 45 s, and extension at 72°C for 45 s, with a final extension step at 72°C for 5 min. The PNA annealing step was adapted from Lundberg et al. (2013). For samples processed without PNA clamps, the same cycling protocol was applied, except that the 78°C PNA annealing step was omitted and the primer annealing at 55°C was shortened to 30 s. PCR reactions were performed in duplicate for each DNA sample in 0.2 mL reaction tubes, resulting in a total of 12 reactions with PNA clamps, 12 reactions without PNA clamps, and four mock controls. Technical PCR replicates were pooled prior to downstream processing. Pooled PCR products were purified using the Gel/PCR Extraction & PCR Clean‐Up Kit (Fisher Molecular Biology), according to the manufacturer's instructions, and eluted in 40 μL of double‐distilled water (ddH_2_O). Amplicon quality was assessed by electrophoresis on 1.5% (w/v) agarose gel. Successfully amplified products (two per time point) were subsequently submitted to Eurofins Genomics for library preparation, using a two‐step PCR protocol, and Illumina amplicon sequencing on a MiSeq platform.

### 2.6 Amplicon Sequencing and Bioinformatic Analysis

Raw sequencing data were analyzed following the pipeline described in Turco et al. (2024) and Cardacino et al. (2025), using the QIIME 2 Docker container (qiime2‐amplicon‐2024.10, Bolyen et al. 2019). Briefly, raw paired‐end reads were demultiplexed, chimeras were removed, denoised through the Divisive Amplicon Denoising Algorithm, and merged (DADA2) (Bolyen et al. 2019). Taxonomic classification was performed using the *classify‐consensus‐blast* algorithm with the SILVA 138 reference database. The resulting feature table, together with the representative sequences and the taxonomy table, was imported into the R environment (v.4.2.3) and further processed using the *phyloseq* (v1.52, McMurdie and Holmes 2013) and *vegan* (v2.7‐1, Oksanen et al. 2025) packages. Bacterial read proportions (bacterial vs. plant reads) were analyzed using a generalized linear model with binomial distribution and logit link function, including method, time point, and their interaction as fixed effects; post hoc comparisons were conducted using estimated marginal means with Bonferroni adjustment, using *emmeans* package (v1.11.2, Lenth and Piaskowski 2026). Alpha within‐sample diversity and evenness were assessed using Shannon and Pielou indexes, after sample rarefaction to the lowest sample size. Beta diversity among samples was assessed via non‐metric multidimensional scaling (NMDS) and Principal Coordinates Analysis (PCoA) based on Bray–Curtis distance. Associations between microbial diversity, method (PNA vs. no PNA), and time points were evaluated using permutational multivariate analysis of variance (PERMANOVA, 1000 permutations) with statistical significance set at *α* = 0.05. Relative abundance was plotted at the genus level using the *ggplot2 f*unction, while Venn diagrams were generated using the *ggVenn* R package to visualize the number of shared and unique ASVs. Differential abundance of bacterial genera was assessed in R using *DESeq2* (v1.48.1, Love et al. 2014) and *ComplexHeatmap* (v2.24.1, Gu 2016) packages. ASVs were agglomerated at the genus level, unclassified taxa removed, and sparse genera (> 90% zeros) filtered out. Analysis included PNA and noPNA samples across TP1–TP3 using the design ~ method + time_point. Size factors were estimated using the poscounts method, and differential abundance was tested with Wald statistics and Benjamini–Hochberg correction (adjusted *p* < 0.05). Significant genera were visualized as row‐scaled heatmaps of relative abundance annotated by method, time point, and phylum.

## 3 Results

### 3.1 PNA Clamps Do Not Alter Bacterial Read Abundance in Mock Community

**FIGURE 1:**
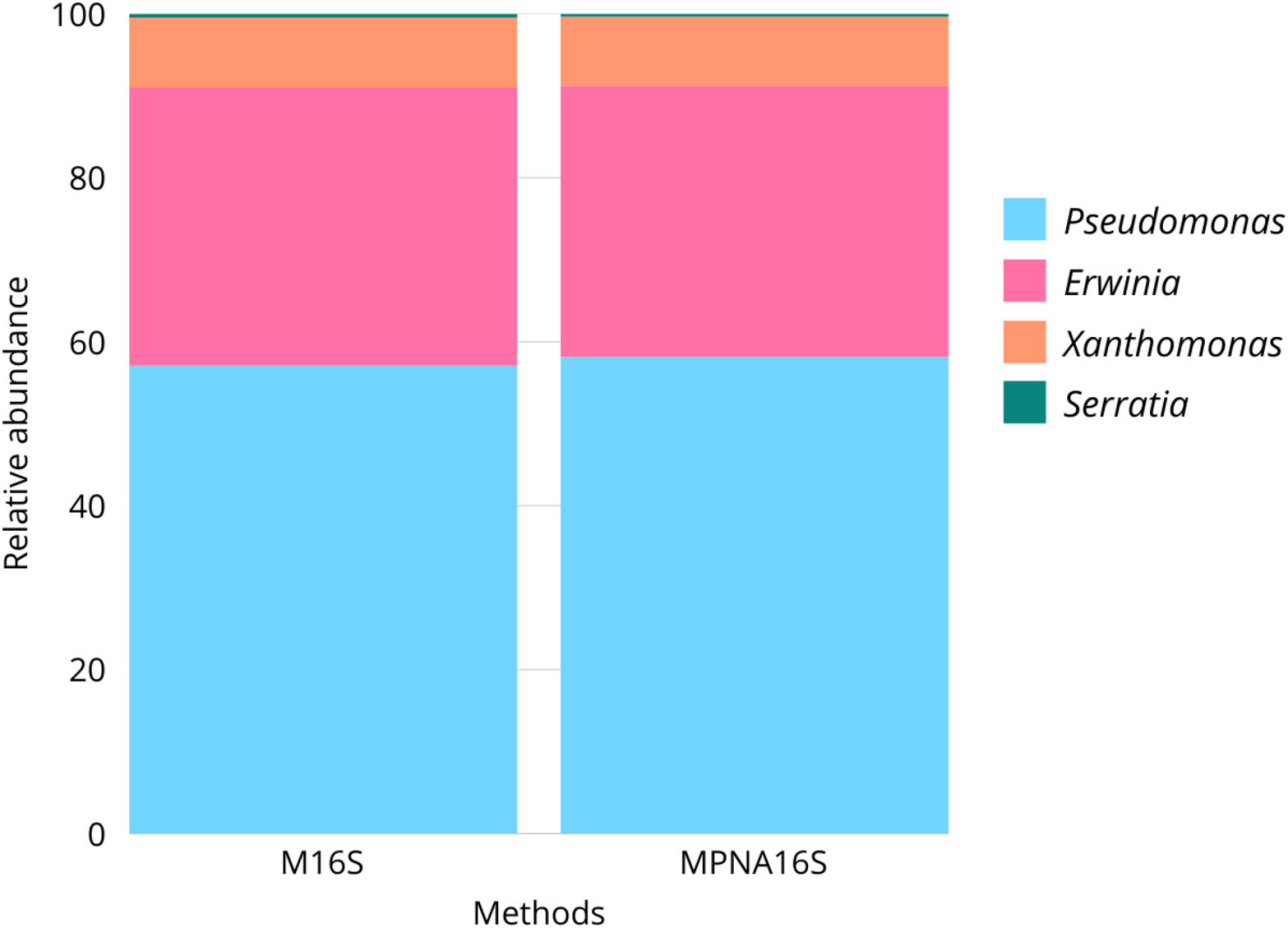
Relative abundances of the four bacterial genera (*Pseudomonas*, *Erwinia*, *Xanthomonas*, and *Serratia*) included in the mock community in samples processed with (MPNA16S) and without PNA clamps (M16S). Comparable abundances across treatments indicate that PNA clamps do not affect bacterial amplification.

To assess the specificity of the PNA clamps, a mock community was sequenced with and without PNA clamps. Relative abundances of the four bacterial genera remained similar between the two conditions, PNA and no‐PNA. Specifically, *Pseudomonas*, *Erwinia*, *Xanthomonas*, and *Serratia* yielded 57.5%, 33.95%, 8.56%, and 0.44%, respectively, in the no‐PNA samples, compared to 58.14%, 33.12%, 8.39%, and 0.35% in the PNA‐treated samples. The observed rates reflected the expected proportions based on the mock community composition, indicating that PNA clamps did not interfere with bacterial amplification and acted selectively against plant‐derived sequences (Figure 1).

### 3.2 Sequencing Results and Reads Proportion

**FIGURE 2:**
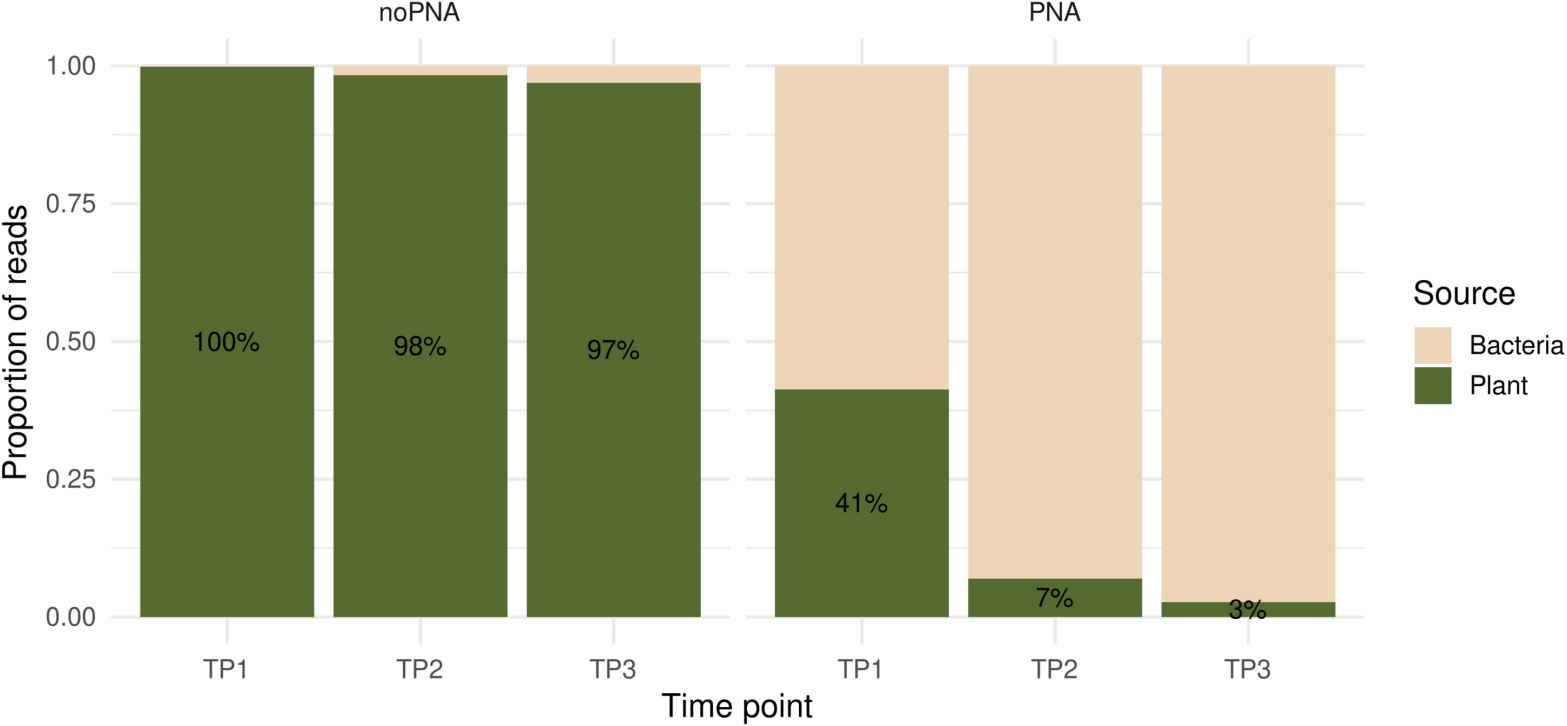
Relative proportions of host‐derived and bacterial reads in samples processed with and without PNA clamps across three seasonal time points (TP1, TP2, TP3). In the absence of PNA clamps, host sequences dominated the datasets, whereas PNA treatment markedly reduced host amplification and increased bacterial read recovery.

Bacterial 16S amplicon sequencing yielded between 50,000 and 150,000 reads per sample (Table S2). Sequencing data were demultiplexed, denoised, filtered for chimeras, and merged, resulting in a total of 920 ASVs across all samples (Table S3), which were reduced to 757 following the removal of plant‐derived ASVs. To assess the effectiveness of PNA clamps in reducing host‐derived sequences and enriching bacterial reads, amplicon sequencing results from samples processed with and without PNAs at three seasonal time points (TP1, TP2, TP3) were compared. Across all time points, samples processed without PNA clamps were dominated by host‐derived sequences, accounting for 100%, 98%, and 97% of total reads in TP1, TP2, and TP3, respectively. Correspondingly, bacterial reads represented only a minor fraction of DNA in a sample (0%, 2%, and 3%, respectively). In PNA‐treated samples, bacterial reads accounted for 59%, 93%, and 97% of total reads at TP1, TP2, and TP3, respectively, while host‐derived reads decreased to 41%, 7%, and 3%. The use of PNA clamps resulted in a reduction of chloroplast and mitochondrial 16S rRNA gene sequences, with a corresponding increase in the proportion of bacterial reads recovered at all sampling time points (Figure 2).

Differences in bacterial read proportions were assessed using a generalized linear model with a binomial distribution. A highly significant effect of method, time point, and their interaction was detected (all *p* < 0.001). Pairwise comparisons with Bonferroni correction confirmed that the proportion of bacterial reads was significantly higher in PNA‐treated samples than in noPNA samples at all‐time points (*p* < 0.001).

**FIGURE 3:**
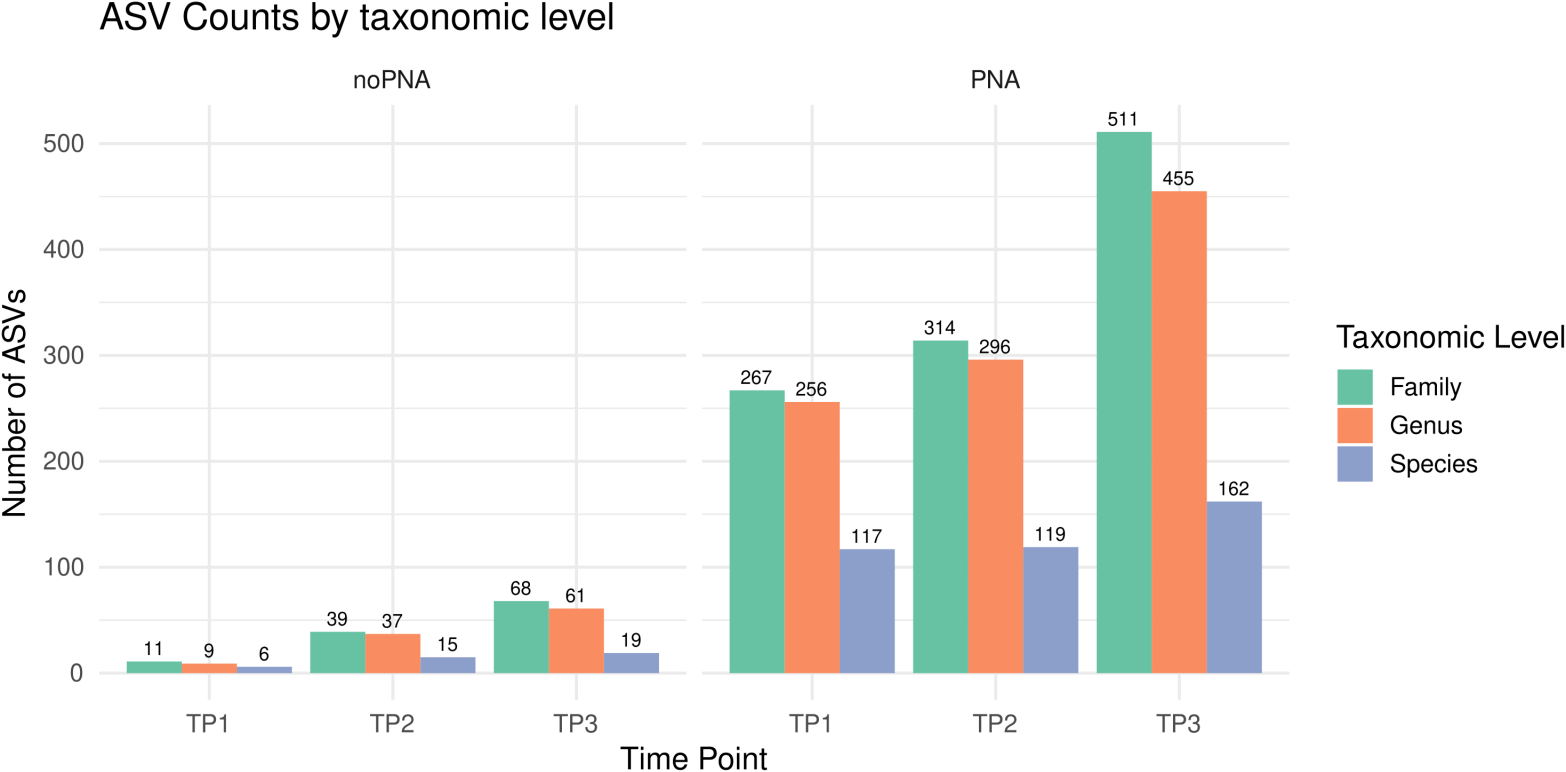
Total ASVs obtained at the family, genus, and species taxonomic levels from samples processed with and without PNA clamps across three seasonal time points (TP1, TP2, TP3).

PNA samples yielded a substantial increase in bacterial reads at all taxonomic levels (family, genus, and species; Figure S1). In contrast, in the absence of PNA clamps, taxonomic resolution was dramatically reduced, primarily due to the low number of bacterial reads. In no‐PNA samples, ASV richness remained consistently low, with only modest temporal increases. At TP1, 11 ASVs were detected at the family level, 9 at the genus level, and 6 at the species level. By TP3, these values increased to 68, 61, and 19 ASVs, respectively (Figure 3). PNA samples exhibited markedly higher ASV counts already at TP1, with 267, 256, and 117 ASVs detected at the family, genus, and species levels, respectively. ASV richness further increased at TP3, reaching 511, 455, and 162 ASVs across the same taxonomic levels.

### 3.3 Leaf Development Drives Temporal Variation in Endophytic Taxonomy

**FIGURE 4:**
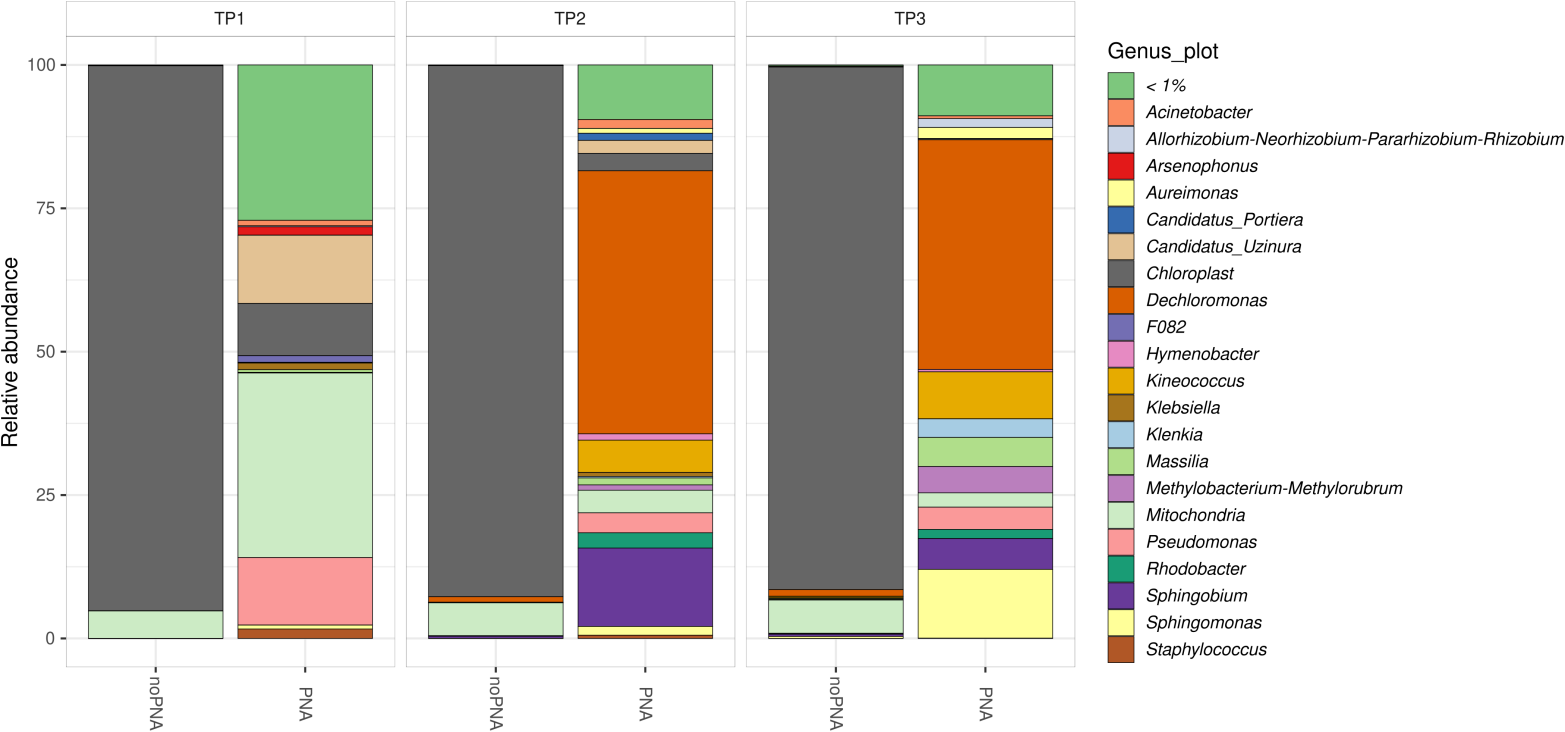
Relative abundance profiles of the most abundant bacterial genera in PNA‐treated samples across three time points (TP1, TP2, TP3). The data show pronounced temporal changes in community composition, with the PNA‐based approach enabling accurate detection of both dominant and low‐abundance taxa by minimizing interference from host‐derived sequences.

According to the previous results, taxonomic classification of the samples processed without PNA clamps revealed the prevalence of mitochondrial and chloroplastic‐associated reads (Figure 4). On the contrary, in samples processed with PNA clamps, *Proteobacteria* were the predominant phylum (up to 278,392 reads), followed by *Actinobacteriota*, *Bacteroidota*, *Firmicutes*, *Patescibacteria*, *Acidobacteriota*, *Myxococcota*, *Gemmatimonadota*, *Bdellovibrionota*, *Chloroflexi*, *Fusobacteriota*, *Campilobacterota*, *Dependentiae*, *Spirochaetota*, and *Cyanobacteria* (Table S3).

Relative abundance profiles at the genus level, restricted to the most abundant taxa, are reported in Figure 4. At TP1, PNA‐treated samples were dominated by *Candidatus Uzinura* (23.3%) and *Pseudomonas* (23.1%). Then, genera such as *Staphylococcus*, *Arsenophonus*, *Klebsiella*, *Acinetobacter*, *Serratia*, *Prevotella*, *Paracoccus*, *Corynebacterium*, *Erwinia*, *Sphingomonas*, *Streptococcus*, *Massilia*, and *Bacillus* followed in a decreasing range from 3.2% to 0.8%. By TP2, the community composition shifted markedly, with *Dechloromonas* (50%) emerging as the dominant genus. Other abundant taxa included *Sphingobium* (14.9%), *Kineococcus* (6.1%), *Pseudomonas* (3.8%), *Rhodobacter* (2.9%), *Candidatus Uzinura* (2.5%), *Sphingomonas* (1.7%), and *Acinetobacter* (1.7%). In addition, several less represented groups were detected, such as *Candidatus Portiera*, *Massilia*, *Hymenobacter*, *Aureimonas*, *Klebsiella*, *Streptococcus*, *Weissella*, and *Erwinia*. At TP3, *Dechloromonas* remained the most abundant genus (42.6%), accompanied by a marked increase in *Sphingomonas* (12.8%). Other consistently represented taxa included *Kineococcus* (8.7%), *Sphingobium* (5.7%), *Massilia* (5.4%), *Methylobacterium–Methylorubrum* (4.9%), *Pseudomonas* (4.1%), and *Klenkia* (3.5%). Additional genera such as *Aureimonas*, *Rhodobacter*, *Leifsonia*, *Acinetobacter*, and *Hymenobacter* were also present at lower abundances, reflecting the broader taxonomic diversity of the community (Table S4). These temporal trends highlight substantial shifts in dominant taxa over time, with the PNA‐based approach enabling high‐resolution detection of bacterial community changes by minimizing host‐derived sequence interference.

### 3.4 Identification of Bacterial Genera Shared Among the Three Time Points

**FIGURE 5:**
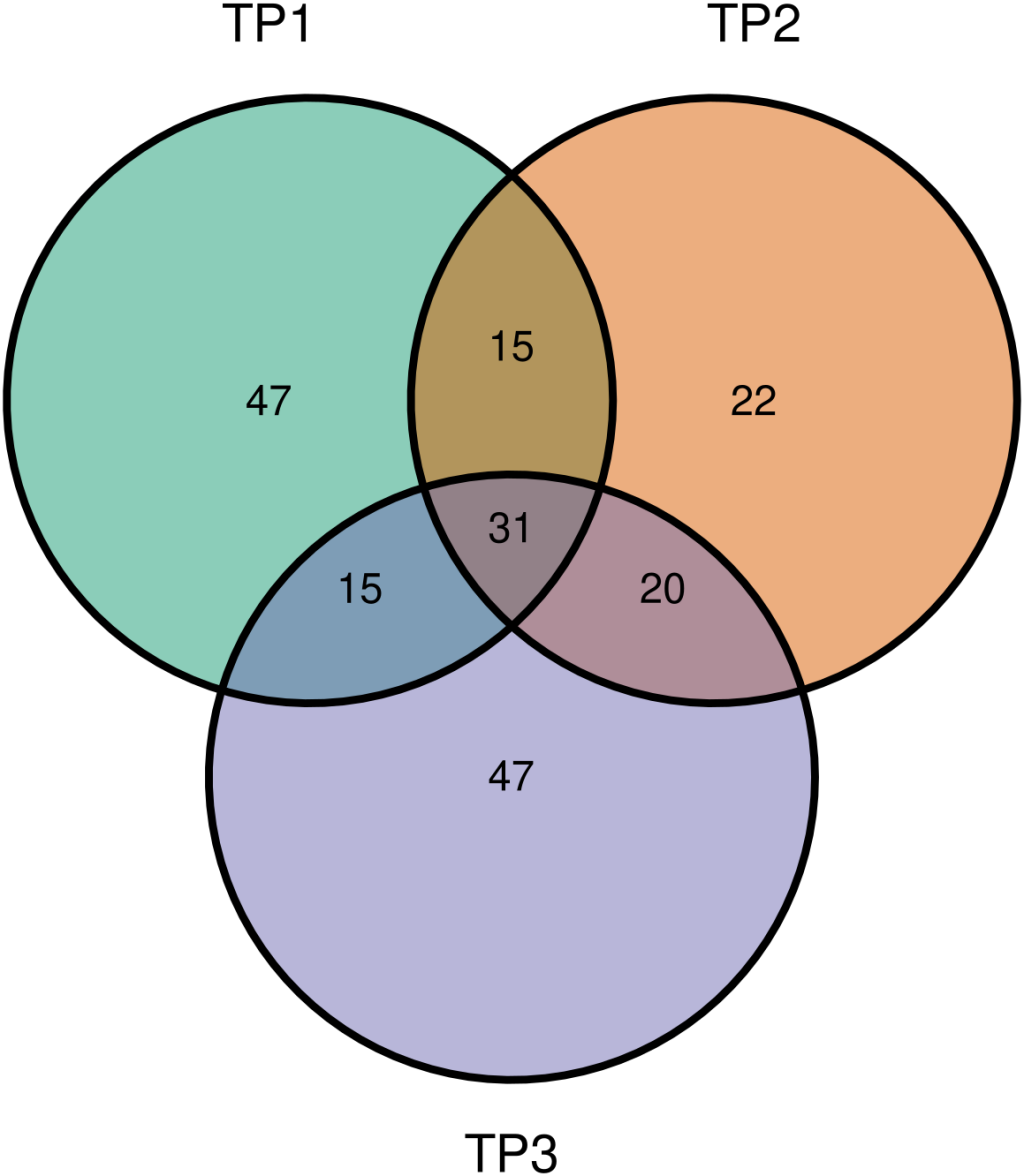
Venn diagrams showing the overlap and unique distribution of bacterial genera detected in PNA‐treated samples across the three time points (TP1, TP2, TP3). The stable core microbiome, comprising 31 genera consistently shared across all time points, was accompanied by unique taxa at each stage (TP1: 47, TP2: 22, TP3: 47), highlighting temporal turnover and dynamic community shifts.

The Venn diagram (Figure 5) shows the overlap and specificity of bacterial groups detected in PNA‐treated samples across the three time points (TP1, TP2, TP3). A core microbiome of 31 members was consistently shared among all time points, including *Pseudomonas*, *Candidatus Uzinura*, *Sphingomonas*, *Staphylococcus*, *Erwinia*, *Acinetobacter*, *Achromobacter*, *Massilia*, *Corynebacterium*, *Bacillus*, *Streptococcus*, *Lactococcus*, *Methylobacterium–Methylorubrum*, *Kineococcus*, *Nocardioides*, and *Hymenobacter*. This stable set of members likely represents a persistent component of the community throughout the sampling period. In addition to the shared core, each time point was characterized by distinct subsets of unique members. TP1 harbored the highest number of exclusive bacterial groups (47), such as F082, *Paracoccus*, *Arsenophonus*, *Candidatus Saccharimonas*, *Brevibacillus*, *Rhodanobacter*, and *Aeromonas*, among others, suggesting a more diverse and transient early community.

TP2 displayed a smaller set of unique members (22), including *Porphyromonas*, *Candidatus Portiera*, *Sodalis*, *Planifilum*, *Comamonas*, *Brachybacterium*, *Hydrogenophilus*, *Sphaerotilus*, and *Aerococcus*, indicating a transitional phase in community structure. TP3 again exhibited a substantial number of taxa (47), such as *Novosphingobium*, *Spirosoma*, *Rhizobacter*, *Frondihabitans*, *Amnibacterium*, *Ralstonia*, *Reyranella*, and *Aeromicrobium*, suggesting the establishment of a distinct late‐stage community composition enriched in members adapted to more specialized or stable ecological niches (Table S5).

### 3.5 Alpha Diversity of Microbial Communities

**FIGURE 6:**
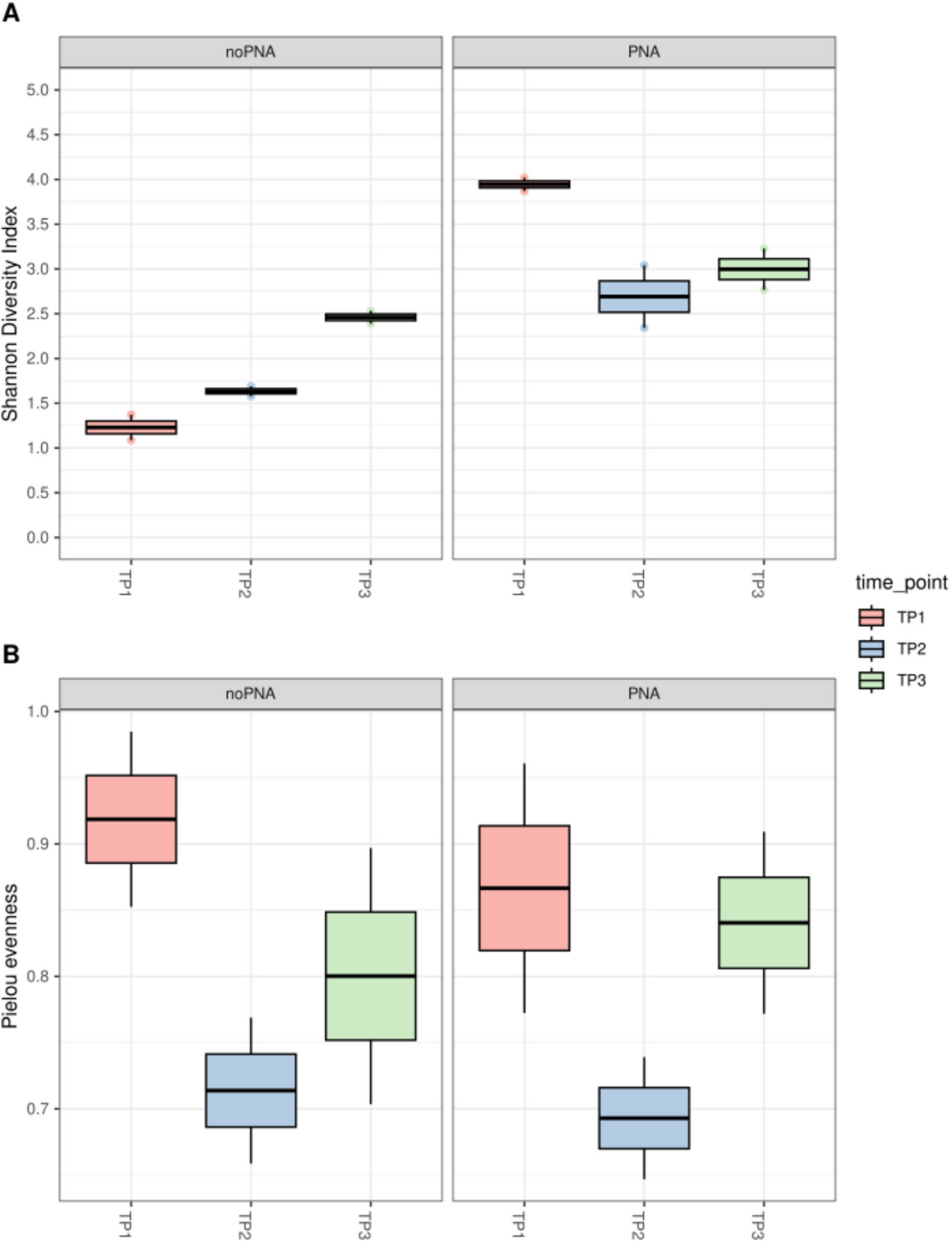
Alpha‐diversity metrics of bacterial communities in PNA‐treated and untreated (noPNA) samples across three time points (TP1, TP2, TP3). Boxplots show Shannon diversity and Pielou evenness.

Alpha diversity analysis revealed clear differences between methods (Figure 6). Shannon diversity, reflecting both taxonomic richness and evenness, was consistently higher in samples processed with PNA compared to those without PNA. The distributions showed limited overlap between the two groups, indicating a strong effect of PNA treatment (Kruskal–Wallis test, *p* = 0.025). In contrast, no significant differences were detected among time points (*p* = 0.50), although temporal variation was visually apparent. In non‐PNA samples, Shannon values appeared to increase from TP1 to TP3, likely reflecting the progressive dilution of host‐derived sequences rather than true ecological dynamics. Conversely, PNA‐treated samples showed a non‐linear pattern, with higher diversity at TP1, followed by a decrease at TP2 and partial recovery at TP3. A similar trend was observed for evenness (Pielou index), with reduced values at TP2 in PNA‐treated samples, suggesting a transient dominance of specific taxa during mid‐season. Overall, these results suggest that, once host DNA interference is minimized, seasonal changes are driven more by shifts in community composition than by changes in overall diversity.

### 3.6 Joint Effects of PNAs and Leaf Development on Microbial Assemblies

**FIGURE 7:**
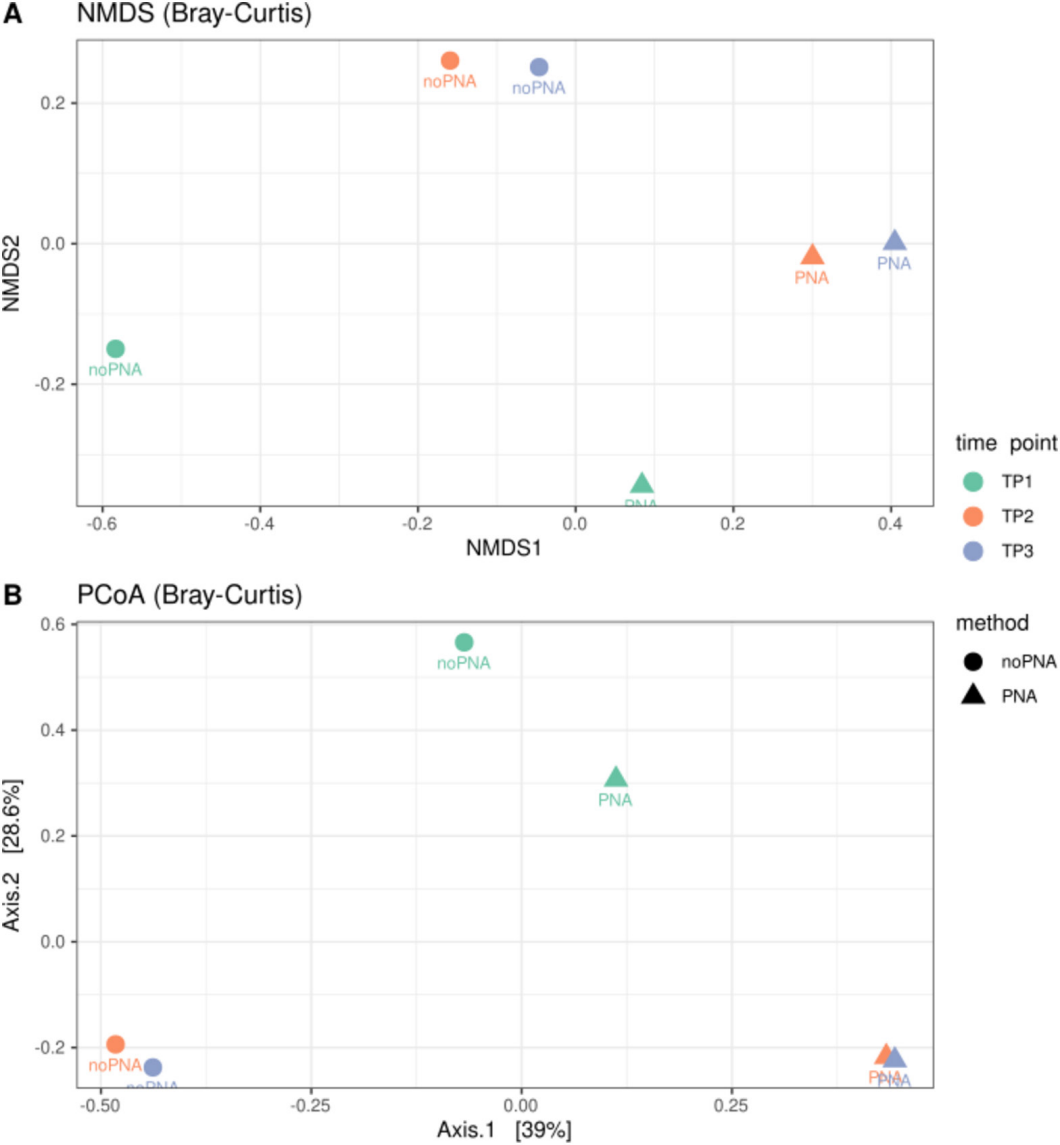
Beta‐diversity analysis of bacterial communities in samples processed with and without PNA clamps across seasonal time points. (A) Non‐metric multidimensional scaling (NMDS) showing that PNA‐treated samples cluster separately from no‐PNA samples at each time point. (B) Principal Coordinates Analysis (PCoA) indicates clear divergence between methods along the primary axis, with seasonal variation contributing secondarily to community structure.

Beta diversity analysis based on Bray–Curtis dissimilarity (Figure 7A,B) revealed a pronounced separation and a statistical difference of bacterial community structures between samples processed with and without PNA clamps (PERMANOVA, *F* = 6.32, *R* ^2^ = 0.31 and *p* = 0.001) across all seasonal time points (PERMANOVA, *F* = 2.88, *R* ^2^ = 0.29 and *p* = 0.008). In the NMDS ordination (Figure 7A), samples consistently clustered according to PNA treatment, with PNA‐treated samples grouping apart from their no‐PNA counterparts at each time point. Similarly, PCoA analysis (Figure 7B) showed clear divergence between the two methods along the primary axis, which accounted for the largest proportion of variation in community composition. While some clustering patterns also reflected seasonal differences (TP1, TP2, TP3), the effect of PNA treatment was the dominant driver of variation. Across both analyses and methods (PNA and noPNA), pronounced differences were observed between T1 and the later time points (T2 and T3), while no significant differences were detected between T2 and T3.

### 3.7 Differential Abundance of Bacterial Genera

**FIGURE 8:**
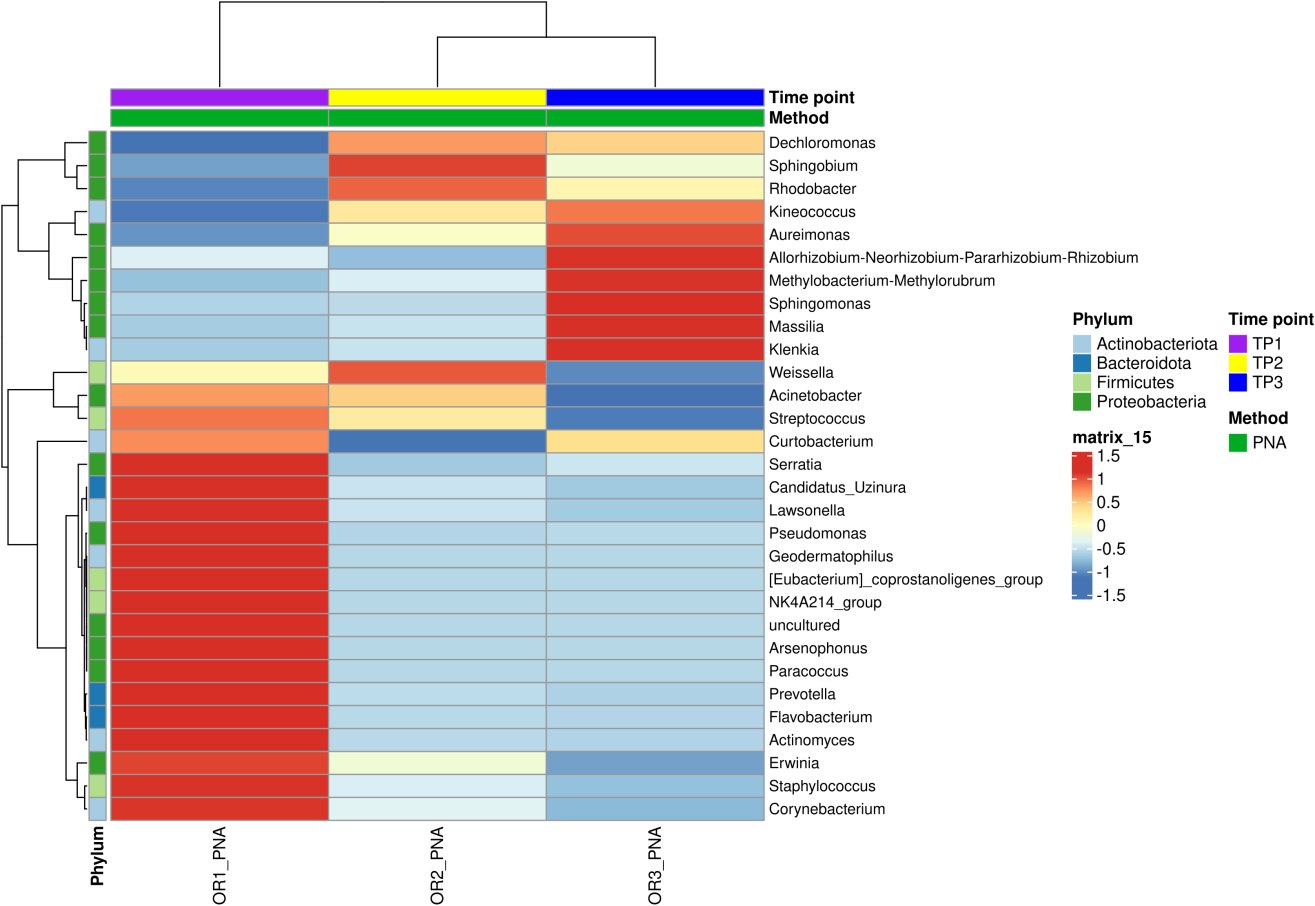
Heatmap showing the differential abundance (DeSeq2 normalized) of bacterial genera across the three time points, based exclusively on PNA‐treated samples. Colors represent relative abundance (red = high, blue = low), with annotations for phylum affiliation, time point, and processing method. Several taxa display clear temporal patterns of enrichment or decline, indicating time‐specific ecological dynamics in the bacterial community.

Differential abundance analysis of bacterial genera across three time points revealed marked temporal dynamics in community composition (Figure 8). Note that only samples processed with PNA clamps were included in the time‐point analysis, minimizing host‐derived sequence interference and enhancing the taxonomic resolution of the bacterial signal. Several taxa exhibited distinct patterns of enrichment or reduction over time, suggesting time‐specific ecological shifts in the bacterial microbiota.

At TP1, the community was dominated by a subset of highly abundant genera, including *Serratia*, *Pseudomonas*, *Paracoccus*, *Erwinia*, and *Staphylococcus*, whose relative abundance significantly declined as early as TP2. In contrast, other genera such as *Dechloromonas*, *Sphingobium*, *Kineococcus*, *Sphingomonas*, *Massilia*, and *Klenkia* showed an opposite trend, with low initial abundance at TP1 followed by a pronounced increase in the later time points, particularly at TP3, indicating a potential successional dynamic as plant–microbe interactions progressed. Most differentially abundant genera belonged to *Proteobacteria*, including both taxa dominant at early stages (e.g., *Pseudomonas*, *Erwinia*) and those enriched later in the season (e.g., *Dechloromonas*, *Sphingobium*). In contrast, *Actinobacteria* were mainly represented by genera increasing toward TP3 (e.g., *Kineococcus*, *Klenkia*). Overall, this pattern suggests that seasonal changes are driven by shifts within dominant phyla rather than by changes in phylum‐level composition.

## 4 Discussion

In this study, characterizing the seasonal dynamics of beech leaf endophytes successfully addressed the major methodological challenge of excessive host‐derived DNA co‐amplification. Our results confirm that organellar (chloroplast and mitochondrial) DNA remains a significant source of bias in beech microbiome surveys, as previously highlighted in broader plant microbiome studies (Bolyen et al. 2019; Hussain et al. 2025).

The high sequence similarity between bacterial 16S rRNA and host organellar regions, a consequence of their shared ancestry, presented a risk of reducing the proportion of informative bacterial reads, potentially obscuring the real structure of the beech‐associated communities (Hanshew et al. 2013). By addressing this similarity, we were able to recover a clearer profile of the leaf endosphere that would otherwise have been dominated by host sequences (Lutz et al. 2011). Universal primers provide broader coverage and higher observed diversity (Hussain et al. 2025), yet are intrinsically prone to organellar co‐amplification (Artimová et al. 2022; Mertin et al. 2025).

Several studies have attempted to mitigate this bias using primer pairs with limited organellar matching (Artimová et al. 2022; Haro et al. 2021; Shi et al. 2023), but these approaches may skew bacterial detection by underrepresenting entire phyla. On the contrary, the use of peptide nucleic acid (PNA) clamps effectively overcame this limitation without reducing taxonomic breadth. The effectiveness of these synthetic DNA analogues lies in their neutral pseudo‐peptide backbone, which confers the high binding affinity and thermal stability necessary to competitively block polymerase extension on beech plastid and mitochondrial templates (Karkare and Bhatnagar 2006; Kawasaki and Ryan 2021; Fouz and Appella 2020; Jasiński et al. 2019). However, as inhibition efficiency may vary by species and organ type, our results provide the required empirical validation for this specific host (Lundberg et al. 2013).

Here, we selected the chloroplast (pPNA) and mitochondrial (mPNA) clamps designed by Lundberg et al. (2013) after confirming 100% sequence identity with *F. sylvatica* organellar 16S regions. Their strong inhibitory effect was experimentally confirmed: libraries not processed with PNAs were dominated by host‐derived sequences (> 90%), whereas PNA‐treated samples yielded an inverted ratio (86%–95% bacterial reads), comparable to previous studies on other plant species (Giangacomo et al. 2021; Hussain et al. 2025). This expanded the number of recovered bacterial ASVs (757), increased richness, evenness, and ecological resolution, while avoiding the loss of rare lineages that bioinformatic host filtering alone may inadvertently discard (Giangacomo et al. 2021; Taerum et al. 2020), and resolved community structure without altering bacterial amplification patterns, as validated in the mock community results (Figure 1). Alpha‐diversity analyses (Figure 6) confirmed that PNA blocking enhanced both richness and evenness, especially at TP1, where organellar DNA interference was maximal. This demonstrates that upstream PNA integration markedly improves the detection of bacterial diversity in low‐yield spring leaves, producing more comparable community profiles through time without amplification bias. Beta‐diversity analysis (Figure 7) revealed that sample separation was dominated by the method effect (PNA vs. no‐PNA), confirming that host organellar DNA is likely to mask ecological signals unless experimentally suppressed (Artimová et al. 2022; Mertin et al. 2025). When focusing only on PNA‐treated samples, communities resolved into a clear seasonal succession, supporting the presence of a real temporal trajectory of the beech leaf microbiome rather than a methodological artifact (Giangacomo et al. 2021; Hussain et al. 2025).

Beech leaves exhibited overwhelming dominance of *Proteobacteria* (76.8% of all PNA‐treated reads, Table S3), in agreement with previous reports from leaf‐associated and endophytic bacterial communities (Compant et al. 2021; Duan et al. 2024; Hardoim et al. 2008). The remaining bacterial reads were distributed across *Actinobacteriota*, *Bacteroidota*, *Firmicutes*, *Acidobacteriota*, and additional phyla detected at substantially lower read counts. In May (TP1), low bacterial recovery (59%) aligns with phyllosphere assembly theory, where communities establish progressively alongside tissue differentiation (Huang et al. 2024; Smets et al. 2022). Venn diagram results supported this seasonal assembly model, revealing a persistent core microbiome of 31 shared genera across all stages, while highlighting 47 exclusive taxa at TP1, 22 at TP2, and 47 again at TP3 (Figure 5). This reflects a structured but flexible microbiome undergoing high taxonomic turnover at the seasonal extremes, whereas summer (TP2) represents a transitional stage dominated by expanding core endophytes, with fewer unique introductions. This turnover at the genus level was further supported by the differential abundance heatmap (Figure 8), which confirms distinct enrichment and depletion profiles at TP1 and TP3.

The predominant genus at TP1 was *Candidatus Uzinura*, an obligate endosymbiont of armored scale insects (Hemiptera: Diaspididae), residing in bacteriocytes and supplying essential nutrients to its host (Gruwell et al. 2012). Although uncommon as a plant endophyte, it has been reported in the phyllosphere of *Tillandsia landbeckii* (Hakobyan et al. 2023) and in olive leaves (Crucitti et al. 2025), where its detection was attributed to sap‐feeding insect communities. A similar ecological interpretation applies to *Arsenophonus*, another early, low‐abundance symbiont of sap‐feeding Hemiptera, known to contribute to host nutrition, defense and reproductive modulation (Dale and Moran 2006; Nováková et al. 2009). Some lineages have been proposed to shift toward plant pathogenicity when insect feeding facilitates bacterial entry into susceptible tissues (Bressan 2014). Its early‐season presence is therefore consistent with transient introduction via insects rather than long‐term colonization. The detection of both taxa likely reflects biological transfer through plant–insect–microbe interactions rather than stable leaf endophytism, a conclusion supported by strict contamination controls during sampling and DNA extraction.

The other bacterial genus that was highly predominant at TP1 was *Pseudomonas*, a broad and heterogeneous taxon that includes lineages able to: (1) stimulate plant growth through synthesis of phytohormones such as indole‐3‐acetic acid (IAA) and through nitrogen fixation (Negi et al. 2023; Suman et al. 2016); (2) sustain foliar iron acquisition via siderophores and improve nutritional balance through phosphorus mobilization (Suman et al. 2016); (3) facilitate internal access and organic substrate turnover through hydrolytic enzymes like cellulases and pectinases (Suman et al. 2016); (4) antagonize pathogens via antimicrobial compounds or niche competition (Martínez‐Rodríguez et al. 2014; Tsalgatidou et al. 2023); and (5) enhance tolerance to drought, salinity, and heavy metals (Schmidt et al. 2018). Although several traits point to a beneficial role (Sharma et al. 2025), pathogenic contributions of some *Pseudomonas* members cannot be fully excluded (Marroni et al. 2024; Porotikova et al. 2025; Purahong et al. 2021).

Beyond early‐season dominants, multiple genera detected across time points are well‐known foliar niche occupants with plant‐supportive functions. *Bacillus* and *Sphingomonas* include lineages repeatedly linked to phytohormone synthesis, improved nutrient uptake, and suppression of pathogens either via antimicrobial metabolites or competitive exclusion (Mushtaq et al. 2023; Ryan et al. 2008). In other phyllosphere models, *Sphingomonas* has been connected to stress modulation and microbial homeostasis (Vega et al. 2005), while *Bacillus* members include disease‐suppressive and nutrient‐enhancing species (Mushtaq et al. 2023; Sharma et al. 2025).

Genera such as *Klebsiella* and *Acinetobacter* may belong to more specialized functional guilds, including nitrogen fixation or the turnover of complex organic substrates, and are often interpreted as contributors to nutrient cycling or abiotic stress tolerance in plant endophytic surveys (Mano and Morisaki 2008). In contrast, *Erwinia* and *Serratia* are less straightforward to interpret, as both genera contain known plant‐pathogenic members. Their detection may reflect the coexistence of strains spanning the mutualism‐to‐pathogenicity spectrum, as expected in microbiome models shaped by environmental or host physiological pressure (Mano and Morisaki 2008).

Additional members more commonly linked to animal or human hosts, including *Staphylococcus*, *Prevotella*, and *Streptococcus*, have nonetheless been observed as plant endophytes, where they are frequently connected with nutrient assimilation, hormone synthesis, or abiotic stress adaptation (Alibrandi et al. 2020; Zhu and She 2018). Of particular interest is the presence of *Massilia*, a genus recognized for its role in environmental remediation, including the degradation of various pollutants, as well as for its plant growth–promoting capabilities. *Massilia* species can enhance nutrient uptake and produce bioactive compounds with protective roles against pathogens (Sedláček et al. 2025). Moreover, they have been reported as components of diverse endophytic assemblages associated with host plant stress tolerance and metabolic adaptation (Li et al. 2025).

*Dechloromonas* showed the most pronounced seasonal expansion from July onward. While its activity in beech leaves has not been directly tested, species in this genus include well‐documented polyphosphate‐accumulating and denitrifying lineages (e.g., “Ca. Dechloromonas phosphoritropha/phosphorivorans”) that are mechanistically linked to phosphorus storage and nitrate respiration (Petriglieri et al. 2021). These pathways align with redox‐active, nutrient‐rich summer microhabitats in leaves. Accordingly, its enrichment is consistent with models in which foliar nutrient turnover/resorption increases during warm, drought‐prone periods (Eckstein et al. 1999), creating niches for bacteria involved in P and N regulation—functions often interpreted as buffering roles in stressed leaf microbiomes (Peñuelas and Terradas 2014). This temporal variation observed at the genus‐level classification underscores the importance of incorporating time‐series perspectives when characterizing plant‐associated bacterial communities.

## 5 Conclusion

This study provides a comprehensive profiling of foliar bacterial endophytes in *Fagus sylvatica* leaves, made possible by suppression of chloroplast and mitochondrial 16S templates through PNA clamps. The integration of the organellar clamps designed by Lundberg et al. (2013) decisively reversed host‐dominated libraries into bacterial‐rich profiles, enabling reliable inference of endophytic community succession from spring to autumn within a single, ecologically distinctive beech stand subject to increasing microclimatic pressure (Mazza et al. 2024).

Several methodological and design considerations inform future work. Marker‐gene amplicon sequencing reflects relative abundances rather than true microbial loads, and the genus‐ and species‐level resolution based on the V3–V4 region remains limited for several bacterial groups, and expanding spatial and interannual sampling will be essential to support broader ecological inference.

From an applied perspective, the identification of distinct seasonal “community states” suggests that specific bacterial taxa or functional categories could be explored as bioindicators for monitoring leaf health in *F. sylvatica* forests. Taxa consistently enriched under mid‐ to late‐season drier and warmer conditions (e.g., *Sphingomonas*) may act as sentinels of microclimatic stress, while early‐season colonizers may reflect successful leaf development and defense priming.

This study, besides confirming the importance of a robust methodological approach in plant microbiome research, lays the groundwork for a clear interpretation of the seasonal endophyte dynamics in beech and broadly covering forest systems.

## Author Contributions

**Irene Giubilei:** writing – review and editing, writing – original draft, conceptualization, validation, visualization, investigation, formal analysis, methodology, data curation, software. **Silvia Turco:** writing – review and editing, writing – original draft, visualization, software, methodology, investigation, formal analysis, data curation, conceptualization, validation. **Antonella Cardacino:** writing – review and editing, formal analysis. **Lovely Mahawar:** writing – review and editing, supervision, resources. **Benedicte Riber Albrectsen:** writing – review and editing, supervision, resources. **Angelo Mazzaglia:** writing – review and editing, writing – original draft, supervision, resources, conceptualization, funding acquisition, project administration.

## Funding

This study was carried out within the Agritech National Research Center and received funding from the European Union Next‐GenerationEU (PIANO NAZIONALE DI RIPRESA E RESILIENZA (PNRR)—MISSIONE 4 COMPONENTE 2, INVESTIMENTO 1.4—D.D.1032 17/06/2022, CN00000022). This manuscript reflects only the authors' views and opinions, neither the European Union nor the European Commission can be considered responsible for them. Since part of this work was conducted at the Umeå Plant Science Centre lab facilities, financial support was also provided by the Wallenberg Foundations.

## Conflicts of Interest

The authors declare no conflicts of interest.

## Supporting information


**Figure S1:** Total bacterial reads obtained at the family, genus, and species taxonomic levels from samples processed with and without PNA clamps across three seasonal time points (TP1, TP2, TP3).

**File S1:** ASV FASTA sequences.

**Table S1:** Mock community taxonomic classification.

**Table S2:** Total number of reads and ASVs count for sample.

**Table S3:** Features table with taxonomic classification.

**Table S4:** Taxonomic classification of the 30 most abundant bacterial genera across the three time points.

**Table S5:** ASVs at the genus level among the three time points.

## Data Availability

The raw data related to this study are available on the NCBI SRA Database under the BIOPROJECT accession number PRJNA1328635.
