# Supplementary figures and images for: Clearing the Noise: Seasonal Dynamics of Endophytic Bacteria in Fagus sylvatica Leaves Revealed by Application of PNA Clamps
Source: Physiol Plant. 2026 Apr 26;178:e70897. doi: 10.1111/ppl.70897 (PMC13110927; doi:10.1111/ppl.70897)

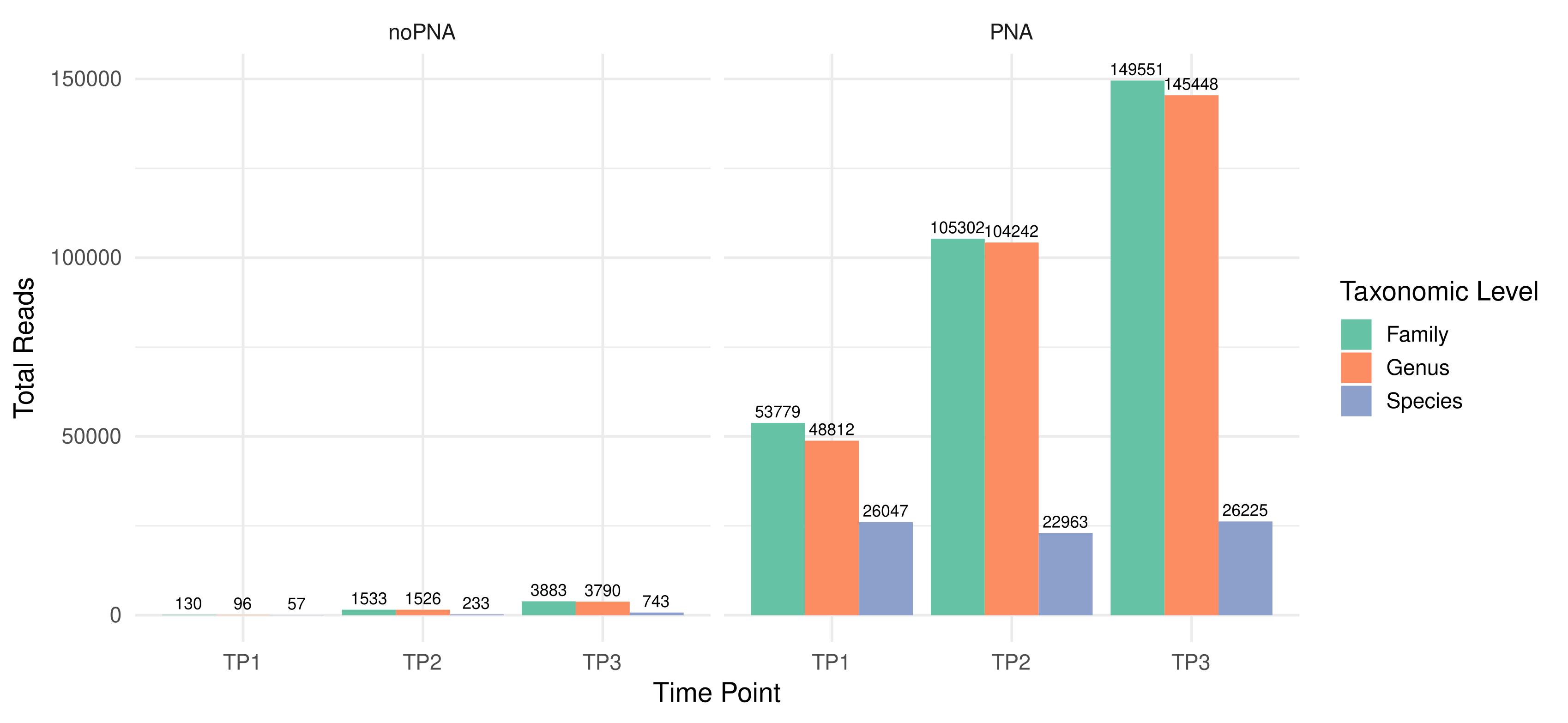

Supplement: Supplementary file 1 — Figure S1: Total bacterial reads obtained at the family, genus, and species taxonomic levels from samples processed with and without PNA clamps across three seasonal time points (TP1, TP2, TP3). [file PPL-178-e70897-s001.tiff]
